# Development of the First WHO Guidelines for Risk Reduction of Cognitive Decline and Dementia: Lessons Learned and Future Directions

**DOI:** 10.3389/fneur.2021.763573

**Published:** 2021-10-26

**Authors:** Ruth Stephen, Mariagnese Barbera, Ruth Peters, Nicole Ee, Lidan Zheng, Jenni Lehtisalo, Jenni Kulmala, Krister Håkansson, Neerja Chowdhary, Tarun Dua, Alina Solomon, Kaarin J. Anstey, Miia Kivipelto

**Affiliations:** ^1^Department of Neurology, Institute of Clinical Medicine, University of Eastern Finland, Kuopio, Finland; ^2^The Ageing Epidemiology Research Unit, Faculty of Medicine, School of Public Health, Imperial College London, London, United Kingdom; ^3^School of Psychology, University of New South Wales, Sydney, NSW, Australia; ^4^Neuroscience Research Australia, Randwick, NSW, Australia; ^5^Population Health Unit, Finnish Institute for Health and Welfare, Helsinki, Finland; ^6^Faculty of Social Sciences (Health Sciences) and Gerontology Research Centre (GEREC), Tampere University, Tampere, Finland; ^7^Division of Clinical Geriatrics, Center for Alzheimer Research, Department of Neurobiology, Care Sciences and Society, Karolinska Institutet, Stockholm, Sweden; ^8^Theme Inflammation and Aging, Karolinska University Hospital, Stockholm, Sweden; ^9^Department of Mental Health and Substance Use, World Health Organization, Geneva, Switzerland; ^10^Institute of Public Health and Clinical Nutrition, University of Eastern Finland, Kuopio, Finland

**Keywords:** dementia, dementia risk reduction guidelines, dementia risk reduction trials, WHO guidelines, tailored interventions, multidomain interventions, cognitive decline

## Abstract

The first WHO guidelines for risk reduction of cognitive decline and dementia marked an important milestone in the field of dementia prevention. In this paper, we discuss the evidence reviewed as part of the guidelines development and present the main themes emerged from its synthesis, to inform future research and policies on dementia risk reduction. The role of intervention effect-size; the mismatch between observational and intervention-based evidence; the heterogeneity of evidence among intervention trials; the importance of intervention duration; the role of timing of exposure to a certain risk factor and interventions; the relationship between intervention intensity and response; the link between individual risk factors and specific dementia pathologies; and the need for tailored interventions emerged as the main themes. The interaction and clustering of individual risk factors, including genetics, was identified as the overarching theme. The evidence collected indicates that multidomain approaches targeting simultaneously multiple risk factors and tailored at both individual and population level, are likely to be most effective and feasible in dementia risk reduction. The current status of multidomain intervention trials aimed to cognitive impairment/dementia prevention was also briefly reviewed. Primary results were presented focusing on methodological differences and the potential of design harmonization for improving evidence quality. Since multidomain intervention trials address a condition with slow clinical manifestation—like dementia—in a relatively short time frame, the need for surrogate outcomes was also discussed, with a specific focus on the potential utility of dementia risk scores. Finally, we considered how multidomain intervention could be most effectively implemented in a public health context and the implications world-wide for other non-communicable diseases targeting common risk factors, taking into account the limited evidence in low-middle income countries. In conclusion, the evidence from the first WHO guidelines for risk reduction of cognitive decline and dementia indicated that “one size does not fit all,” and multidomain approaches adaptable to different populations and individuals are likely to be the most effective. Harmonization in trial design, the use of appropriate outcome measures, and sustainability in large at-risk populations in the context of other chronic disorders also emerged as key elements.

## Introduction

The number of people with dementia is projected to triple within the next 30 years (1). The lack of an effective treatment has an ever-increasing impact on public health and highlights the importance of effective prevention strategies. In 2019, the publication of the first World Health Organization (WHO) guidelines for the risk reduction of cognitive decline and dementia marked an important milestone in this context (2).

The guidelines were developed during 2018 based on the evidence available at the time on twelve modifiable risk/protective factors identified within recent systematic reviews (SRs) (1, 3, 4) and guidelines (5): physical activity; tobacco consumption/cessation; nutrition; alcohol use disorders; cognitive activity; social activity; overweight/obesity; hypertension; diabetes mellitus; dyslipidaemia; depression; and hearing loss. The guidelines are mainly based on clinical trial evidence from interventions targeting these factors (2) and recommendations were formulated for 10 of the 12. A lack of trial evidence resulted in additional or alternative observational evidence sourced for several of the factors, and insufficient evidence was reported in relation to *social activity* and *hearing loss*, preventing the definition of specific recommendations.

The aim of this paper is to critically discuss the main themes that emerged from the evidence review and based on this, to inform the most effective directions for future research on dementia risk reduction. The future development of more robust evidence for the best strategies for dementia risk reduction, together with new evidence reported since the publication of the guidelines, will help refine the recommendations in the next update of the guidelines, not only for factors already included, but also by potentially including new factors and, thus, improve public health policies aimed at dementia prevention.

## Main Themes Emerged From the Review of the Evidence

The systematic review and quality assessment of the evidence was based on the “Grading of recommendations, assessment, development, and evaluations” (GRADE) methodology (6). The highest level of evidence was provided by systematic reviews (SRs) of randomized controlled trials (7, 8) (RCTs), whereas single RCTs or SRs of observational studies were considered as a lower level evidence and included when the highest level of evidence could not be identified or were not deemed sufficient (2).

The present section describes the key common themes that emerged during this process and advises on the most effective future directions in dementia risk reduction research. In Supplementary Table 1, explanatory examples of evidence included in the Guidelines are presented together with more recent relevant studies. S*ocial activity* and *hearing loss* are not included since recommendations could not be formulated. However, further results from RCTs are expected for hearing loss (9) and more SRs related to *social activity* (10, 11) have been available since the publication of the guidelines.

### Intervention Effect-Size

#### Effect-Size in the Context of Population-Size

Overall, the effect-sizes reported by RCTs are relatively small [Supplementary Table 1; e.g., for *nutrition* the effect of the Mediterranean diet compared to control was reported as standard mean difference = 0.24; 95% CI: 0.00–0.47; (12)]. However, as the “prevention paradox” for cardiovascular disease (13) (CVD) suggests, effect-sizes must be considered in the context of overall health benefits and target population. Interventions with small dementia risk reduction effects could, indeed, lead to a large-scale change when implemented in a wide lower-risk population, rather than a small high-risk group. For example, although aerobic exercise interventions in cognitively normal older adults had a comparable effect-size to similar interventions in people with Mild Cognitive Impairment (MCI) (Supplementary Table 1), the former could have a much bigger overall impact, especially in a public-health context, since a much larger population can benefit from it.

#### Effect-Size in Lifestyle vs. Pharmacological Interventions

The evidence gathered mostly concerns lifestyle interventions, which could have smaller effect-size compared to pharmacological ones. Although for *diabetes* prevention it has been suggested that lifestyle interventions could be at least as effective as pharmacological treatments (14), in other cases, e.g., cardiovascular disease (CVD), lifestyle-based approaches could have smaller individual impact than pharmacological treatments. However, their potential advantages, e.g., lower/milder side effects, make lifestyle interventions a more reachable “low hanging fruit,” bearing a smaller individual effect but bigger benefits in larger populations, provided that the lifestyle intervention is well-accepted and sustainable.

#### Single-Domain Approach

Interpretation of the effect-size of the intervention included in the guidelines must also take into account that these trials target one single risk factor at the time. Multidomain interventions targeting multiple factors simultaneously have the potential to produce greater benefits. However, their efficacy, especially in relation to single-domain ones, cannot be interpreted based on a simple comparison of effect-sizes. Effect-size in the context of multidomain interventions is discussed in a later section.

### Mismatch Between Observational and Intervention Evidence

Various degrees of mismatch between observational and trial evidence have been observed across risk/protective factors. For some factors (e.g., *overweight/obesity* and *depression*, Supplementary Table 1) the exposure-outcome associations in observational studies are bigger than the effect of intervention trials. The SR of RCTs aimed at weight reduction (15), reported, at most, a standard mean difference of 0.44 (95%CI 0.26–0.44) favoring the intervention vs. control in the attention cognitive domain, and no significant difference in any other cognitive domains considered. On the other hand, the observational evidence focusing on the association between *overweight/obesity* at midlife and incident dementia (RR = 1.44; 95% CI: 1.20–1.60) (16) suggest a much more relevant role for this risk factor.

In other cases, relatively large associations in observational studies did not translate into any significant intervention benefit in RCTs (Supplementary Table 1) and the results reported for *dyslipidaemia* are a typical example. Although static use was found to be significantly associated with decreased risk of dementia (RR = 0.62; 95% CI: 0.43–0.81) in observational studies (17), no significant intervention benefits have been identified, so far, in RCTs (OR = 1.00; 95% CI: 0.61–1.65) (18). In more extreme cases (e.g., *tobacco consumption/cessation* and *alcohol use disorders*) a substantial body of observational evidence contrasted with a total lack of evidence from RCTs, partly due to the ethical issues posed by potential randomization to risk factor exposure or placebo/control.

The underlying causes of such mismatches are not fully clear. One key clue could lie in the duration of observational studies, which can span a much longer period of time, compared to RCTs. This can potentially affect not only the size of the association/effect reported but, more importantly, determine the choice of feasible outcomes. Clinical endpoints for slowly developing dementia are routinely used in longitudinal studies (especially with long follow-ups), but rarely achievable in the timeframe of RCTs. Cognition assessed through standardized tests is the preferred surrogate option, but the application of other indirect risk-estimation tools, such as well-established risk scores, could also be effective (19, 20) and should be explored more systematically.

The only clear exception to the mismatch between observational and RCT evidence was *hypertension*, for which RCT evidence broadly aligns with observational data (Supplementary Table 1). In particular, the most recent SR of RCTs (21) reported an odd ratio for dementia/cognitive impairment of 0.93 (95% CI: 0.88–0.98) for pharmacological interventions aimed to reduce blood pressure, in line with the most recent observational evidence (22) on the association between use of antihypertensive and AD diagnosis (RR ranging 0.71–0.92; 95% CI ranging 0.59–0.83 to 0.79–1.08).

### Heterogeneity of Evidence in Meta-Analysis of RCTs and Observational Studies

Significant heterogeneity among RCTs or observational studies, formally assessed through *I*^2^ or *Q* statistics, is a relatively common feature of the evidence included in the Guidelines [Supplementary Table 1, e.g., *I*^2^ = 60% in SR of RCTs for *overweight/obesity* (15); *I*^2^ = 90.5% in SR of observational studies for *cognitive activity* (22); *I*^2^ = 70.8% in SR of observational studies for *dyslipidaemia* (17)]. Although methodological aspects (e.g., inclusion/exclusion criteria) of the SRs can play a role, the most plausible explanation is the marked differences in study and intervention design among individual RCTs included in the SRs and meta-analyses considered in the development of the guidelines. The evidence that formed the foundation for the recommendations for *overweight/obesity* (15) is a clear example in this sense. A wide range of intervention strategies aimed at weight reduction were included (alone or combined): diet, physical activity, calorie restrictions, increased unsaturated fatty acids. Furthermore, even when the same approach was applied, the specific content (e.g., type of diet/exercise, duration, inclusion criteria) would differ among trials, contributing to different levels of weight loss.

Lack of consistency in the design of studies addressing relatively similar questions represents an important limitation, not only for traditional meta-analyses, but also when pooling data at individual level to power explorative and novel analysis (23). Harmonization of RCTs protocol and intervention designs should, therefore, become a higher priority.

### Intervention Duration

Intervention duration is crucial when interpreting the efficacy of an intervention. Implementation of sustainable healthy habits that can be maintained after the end of the more intensive trial period is particularly important for lifestyle-based interventions, for which long-term less-intensive strategies could provide the best results. A dilution effect for longer interventions compared to shorter ones has been reported, e.g., cognitive training (24), with decrease of adherence during longer interventions being one of its possible explanations. However, its causes have not been elucidated and it would not necessarily apply to all interventions. Therefore, it is still plausible to assume that, overall, the effect of public-health interventions based on sustained long-term behavior changes in larger population have the potential to increase over time.

### Time Is Everything

Timing of exposure and intervention was highlighted as a key factor in the evidence synthesis.

Several risk/protective factors play a different role in the dementia risk based on age. Some, like *dyslipidaemia, overweight/obesity*, and *hypertension*, are reportedly more relevant in mid-life (4). For example, the observational evidence for *overweight/obesity* (16) reported associations with either an increased (RR = 1.41; 95% CI: 1.20–1.66) or a decreased (RR = 0.83; 95% CI: 0.74–0.94) risk of dementia depending on whether the risk factor had been investigated at midlife or at a later age, respectively (Supplementary Table 1). Other risk factors, such as *physical inactivity* and *diabetes*, are more often found to be associated with dementia risk in late-life (4). This can crucially inform as to the best target population and most effective intervention design.

#### The Role of Reverse Causality

Reverse causality has been proposed to explain the differences in risk factor significance across the lifespan (25–29), casting uncertainty on the utility and safety of certain interventions, especially in late-life. Evidence on the changes in the role of risk factors at different stages of life is mostly based on observational studies, and its relevance for RCTs is not yet fully understood. *Physical inactivity*, for instance, could be, in late life, a consequence of an ongoing neurodegenerative process (25). However, evidence showed that *physical activity* can still be modified in older adults through controlled and safe interventions that still have the potential to improve fitness and cognitive status (30). Therefore, when targeting risk/protective factors potentially linked to reverse causality, desirable and undesirable intervention effects should be weighted systematically, as done in the guidelines development (2), and the target population should be carefully chosen based on realistically achievable lifestyle changes.

#### When to Start

The right starting time is also central to the success of an intervention. Based on the evidence collected, it is not clear at what stage of the disease continuum a certain intervention can deliver the greatest benefits. Interventions for *physical activity* including at-risk cognitively normal people (30) were more effective than those targeting MCI (31), suggesting that even small cognitive impairment can hamper their efficacy, although RCTs can vary in terms of duration, intensity, sample size, and study design in general. However, older adults without cognitive impairment can have different risk profiles characterized by the presence of different risk/protective factors, thus the best window of opportunity for prevention may not be the same for everyone.

### Dose-Response Relationship

Evidence of dose-response relationship is very scarce both in observational studies and RCTs. Some indications were found for *alcohol use disorders*. The lowest risk was associated with 6 g/day and the association between alcohol consumption and dementia risk was significant from 38 g/day (32). For *tobacco consumption*, an increased dementia risk by 34% was reported for every 20 cigarettes per day (33). Attempts were also made to define dose-response relationship for more complex factors, such as *physical activity* (34) and *nutrition* (dietary patterns, in particular) (35), but were limited by inconsistencies in exposure reporting and lack of data from RCTs. For observational data, there has been an indication of linear dose-response relationship between adherence to dietary pattern and risk of any cognitive disorder, but the results were still non-significant and definitions of high vs. low dose vary between the studies, therefore no definite conclusions can be drawn. Finally, antihypertensive treatments seem as effective regardless of starting BP (36, 37), although there are likely to be limits and a specific dose-response relationship with dementia risk reduction is not fully clear.

As proposed for CVD (38), a threshold effect, whereby a certain minimum amount of change is required to achieve any benefit, is likely to be applicable also to dementia risk reduction, suggesting that doing the “right” thing is not enough, but doing “enough” of it is also necessary. Thus, identifying the optimal intensity level for a certain intervention is a crucial prerequisite to optimize and harmonize study design.

### Link to Specific Dementia Pathologies

Most of the systematic reviews considered do not discriminate among dementia pathologies and disease specific studies focus consistently on Alzheimer's Disease (AD). Additionally, many studies consider primarily clinical diagnosis of different dementia types. Some evidence has been collected from observational studies for *tobacco consumption* and *nutrition*. The association between smoking and risk of AD or vascular dementia (VaD) has been inconsistent (33) although increased risk of vascular brain injury, global and hippocampal atrophy has been reported (39). Observational studies also suggested that Mediterranean diet (MeDi) could be more strongly associated with AD than VaD or any dementia, based on a specific association of MeDi with amyloid accumulation and brain atrophy (40, 41), but more evidence is needed from longitudinal studies. Mixed brain pathologies are extremely common (42) and represent a major challenge for these investigations.

The evidence linking risk/protective factors to individual pathologies is, in general, incomplete. Several biological mechanisms have been proposed for dementia risk factors (e.g., vascular and metabolic related mechanisms, inflammation, oxidative stress), but their potential links with specific brain pathologies have not been fully elucidated. Until recently, non-pharmacologic RCTs only sporadically included assessment of biomarkers such as cerebrospinal fluid or brain imaging (43).

From a preventive perspective, identifying the strategies that work best could be deemed more important than understanding exactly how they work. Moreover, recommendations for dementia risk reduction could still be developed based on available evidence. However, in the long-term, research would considerably benefit from elucidation of the links between individual risk factors and specific brain pathologies. This is particularly relevant for those factors (e.g., *social engagement, hearing loss*, and, to a certain extent, *overweight/obesity*) that are thought to act as proxy for others (e.g., *cognitive stimulation, diabetes*) that are more tightly linked with specific biologic mechanisms of neurodegeneration. In these cases, a better understanding of the link with specific brain pathologies could provide key information on biologic mechanisms and the best preventive/therapeutic targets.

### The Need for Tailored Evidence and Interventions

The review of RCTs and observational studies has pointed to a lack of evidence targeted to particular risk groups, either by virtue of age, region, cultural background, genetic risk profile, or other factors. For example, despite *dyslipidaemia* being highlighted as a potential risk factor mostly in midlife, trials testing the efficacy of statin treatment in reducing dementia risk were conducted in older adults (44). Although justified by the time constraints of an RCT and the need to target a population at increased risk of developing cognitive decline within a relatively short timeframe, this represents a major limitation and a possible reason for the failure of such trials. Additionally, most RCTs are still conducted in the United States or Europe and include few minority groups (22), making their results unlikely to be applicable in diverse societies. Yet, some factors can vary considerably by cultural (e.g., *nutrition*) or ethnic (e.g., *diabetes*) group and diverse population subgroups may respond differently to differing patterns of risk factor exposure and interventions (45). More tailored approaches have the potential to be more effective.

## Interaction and Clustering of Risk Factors

In the general population, the risk factors considered here are unlikely to be fully independent from each other and to occur in isolation, not only in single individuals, but also in specific geographical locations and population groups. Identification of different patterns of risk/protective factors among individuals or population subgroups represents an opportunity for more tailored approaches and may have a proportionately greater impact than generic public health messaging. Whilst there is relatively little data on risk factor clustering and risk of later dementia or cognitive decline, multiple studies have attempted to assess the combined impact of concomitant risk factors for dementia, using scoring paradigms where greater numbers of risk factors (with or without some weighting) result in higher risk scores. Eighteen studies were identified in a SR examining the impact of comorbid modifiable risk factors for dementia (46). Despite the studies drawing from diverse populations and reporting varied lengths of follow-up, a near universal increased risk of dementia was identified with the addition of each modifiable risk factor. The meta-analysis of the studies with long follow-up and dementia as outcome confirmed this finding. This is particularly interesting since all studies included different combinations of risk factors, but no risk factor was present in all studies, implying that the number of risk factors might be at least as important as the specific risk factor. This suggests that multidomain interventions targeting concomitant lifestyle and clinical modifiable risk/protective factors may be necessary to achieve a meaningful impact. This, however, does not automatically imply that only an additive effect is at work, since interaction and clustering of multiple risk and protective factors may also have a role to play and the effects of multidomain interventions do not reflect the mere sum of the effects observed when one risk/protective factor is addressed at a time. As shown previously in coronary heart disease (47) and type 2 diabetes (14) prevention, simultaneous smaller, longer-term changes in several risk factors can, indeed, lead to important protective effects and, especially in a public health context, where people have a wide range of risk factor severity and profiles, this approach could be more effective and safer than radical short-term changes in a single risk factor.

Finally, the findings of the cumulative impact of increasing number of risk/protective factors raises the question of whether specific combinations of medical conditions or health behaviors have different long-term impacts on the risk of dementia. In addition, specific combinations of environmental and genetic (APOE, in particular) risk factors may affect the risk of dementia. For example, the investigation of whether the beneficial effects of physical activity interventions on cognition are modified by APOE genotype is ongoing. Understanding the interactions among risk factors will lead to better targeted intervention. Multidomain interventions can also be implemented with a higher degree of flexibility and, therefore, more easily tailored to single individuals or specific target populations, based on their risk factors patterns, needs and lifestyle.

## Interactions Between Genetics and Environmental Risk Factors

Population attributable risk prediction models suggested that about 40% of dementia cases are attributable to modifiable factors (48) and 7% to apolipoprotein E (ApoE) ε4 allele, the major genetic risk factor for AD (4).

Given the multifactorial etiology of dementia, interactions between genetics and combinations of environmental (i.e., non-genetic) factors can potentially provide more relevant insights for risk reduction strategies. However, studies focusing on individual factors are much more common (49). In this regard, our current knowledge is mainly based on observational studies, with only limited evidence available from RCTs, and the findings are far from conclusive.

Within the Cardiovascular Risk Factors, Aging, and Incidence of Dementia (CAIDE) study, the late-life risk of dementia was reported to increase in ApoE-ε4 carriers with an unfavorable (non-genetic) risk profile at midlife (50). Recently, two large observational studies have investigated the interaction between genetics and environmental risk factors, modifiable, and lifestyle-related, in particular (51, 52). In both cohorts it was reported that a combination of unhealthy lifestyle (defined through e.g., smoking status, physical activity, diet, and alcohol consumption) and high genetic risk was associated with a higher risk of dementia compared to healthy lifestyle and low genetic risk. However, while in the first study people benefitted from a healthy lifestyle in terms of dementia risk reduction regardless of the genetic risk (52), such protective associations were not identified for individuals at high genetic risk in the second (51).

The importance of risk profiles, especially at midlife, has been explored in detail over the past years. Risk modification at midlife has been shown to be associated with lower risk of cognitive decline and dementia (46), but it is unclear what role the genetic risk plays in this context. The effect of ApoE-ε4 is thought to attenuate with increasing age (53) and dementia onset in ApoE-ε4 carriers occurs at an earlier age compared to non-carriers (54). Therefore, in ApoE-ε4 carriers in particular, it could be sensible to intervene with risk modification strategies at midlife, or even earlier, before the onset of reverse causality mechanisms, and whilst benefits from genetics-lifestyle interactions are potentially still achievable.

In the context of multidomain interventions, the Prevention of Dementia by Intensive Vascular Care (preDIVA) trial (55), the Finnish Geriatric Intervention Study to Prevent Cognitive Impairment and Disability (FINGER) (56), and the Multidomain Alzheimer Preventive Trial (MAPT) (57) have investigated the modifying effect of ApoE status in response to the intervention. Although interaction analyses in all trials showed that ApoE status did not affect the overall response to the intervention, beneficial effects were found for the FINGER and MAPT interventions in ApoE-ε4 carriers in stratified analysis.

More evidence is needed from multidomain RCTs to elucidate the complex interaction between genetics and environment and help define the most suited target population also based on genetic risk.

## Multidomain Trials and Contextual Approaches

### Review of Multidomain Trials

**Figure 1:**
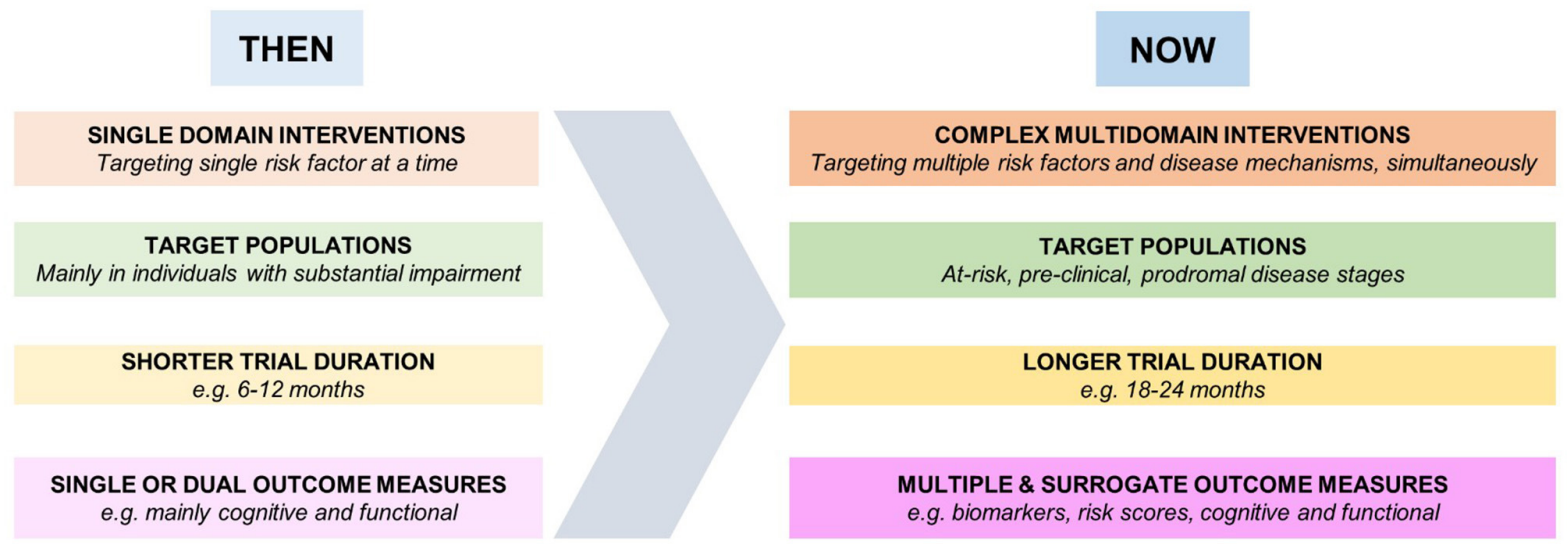
Development of dementia risk reduction trials.

Given the recent scientific advances in diagnostic criteria (58) and lessons learned from previous RCTs (59), the design of RCTs aimed at dementia risk reduction has considerably changed in recent years (Figure 1). Target populations with the highest potential for improvement have been mostly identified at the early stages of the disease continuum, with profiles ranging from at-risk and/or asymptomatic individuals to people with minimal cognitive deficits. The trial duration has also increased, recognizing the potential and the importance of long-term effects. Recently, increasing interest has been shown toward integrated approaches, either combining multiple pharmacological therapies (60) or targeting the same lifestyle factor through a multicomponent intervention.

This includes, for example, multi-nutrient interventions (61); interventions for physical activity combining aerobic, resistance, and multimodal training (62, 63); and cognitive training trials, combining various social, and intellectual activities (64, 65).

More recently, a newer generation of multidomain trials has been established (Table 1).

**Table 1:** Summary of non-pharmacological multidomain (at least three components) trials with cognitive decline related primary outcomes.

**Study** **(references)**	**Design and population**	**Multidomain intervention**	**Primary outcome**	**Main results**
ASPIS (66)	*N* = 202 Stroke patients Age: 40–80 years Duration: 2 years	Multidomain intervention (clinical therapy, adequate blood pressure, lipid and glycaemic control, healthy diet, regular physical activity, cognitive training) vs. standard stroke care	Cognition on Alzheimer's Disease Assessment Scale and neuropsychological test battery	No difference between intervention and control groups
FINGER (56)	*N* = 1,260 Persons at-risk of dementia Age: 60–77 years Duration: 2 years	Lifestyle intervention (diet, exercise, cognitive training, vascular risk monitoring) vs. general health advice	Cognition on neuropsychological test battery	Significant improvement in cognition in the intervention group compared with the control
Pre-DIVA (55)	*N* = 3,526 Community-dwelling older persons Age: 70–78 years Duration: 6 years	Multidomain intensive vascular care vs. standard care	Disability score and incident dementia	No difference between intervention and control groups
MAPT (57)	*N* = 1,680 Community-dwelling older persons Mean age: 75.3 years Duration: 3 years	1. Multidomain intervention + omega-3 supplementation vs.2. Multidomain intervention + placebo vs.3. Omega-3 supplementation alone vs.4. Placebo alone	Cognitive decline on composite *Z* score	No difference between intervention and control groups
KENKOJISEICHI (67)	*N* = 83 Individuals with MCI Mean age: 76 years Duration: 24 weeks	Physical, cognitive, social activity sessions vs. health education	Cognition on National Center for Geriatrics and Gerontology Functional Assessment Tool	Significant intervention benefits on spatial working memory compared with the control group
BBL-C (68)	*N* = 119 Individuals with subjective cognitive decline or MCI Mean age: 73.0 Duration: 8 weeks	Diet, exercise, and cognitive training sessions + online educational modules vs. online educational modules only	Cognition on Alzheimer's Disease Assessment Scale and neuropsychological test battery	Significantly higher cognition score in the intervention group compared with the control group

*ASPIS, Austrian Polyintervention Study to Prevent Cognitive Decline After Ischemic Stroke; BBL-CD, Body Brain Life for Cognitive Decline; FINGER, Finnish Geriatric Intervention Study to Prevent Cognitive Impairment and Disability; MAPT, Multidomain Alzheimer Preventive Trial; preDIVA, Prevention of Dementia by Intensive Vascular Care*.

The preDIVA trial tested a 6-year multidomain intervention in an unselected primary care population of older adults (55). No significant effects were reported for the primary outcomes. However, although the intervention reduced systolic blood pressure (69) and benefits were observed in participants with elevated and untreated blood pressure at baseline and that initiated antihypertensive treatment as part of the intervention (55), no beneficial effect was reported for any of the other risk factors considered (69).

The MAPT 3-year multidomain intervention did not lead to significant effects on the primary outcome. However, *post-hoc* analyses showed beneficial effects of the multidomain intervention regardless of supplementation status or on individuals with an increased risk of dementia [i.e., participants with a CAIDE score of 6 or more; (57)].

The Austrian Polyintervention Study to Prevent Cognitive Decline after Ischemic Stroke (ASPIS), compared the efficacy of a 2-year intensive multimodal lifestyle intervention in patients with ischemic stroke vs. standard stroke care in reducing the risk cognitive decline. No effects were observed on the primary outcomes (66), which could be due to small sample size, inclusion of middle-aged adults, and/or inefficacy of the multidomain approach in post-stroke cognitive decline.

The KENKOJISEICHI trial tested a 24-week intervention combining physical, cognitive, and social activity against health education in 83 people with MCI. The primary outcome was cognitive function and significant intervention benefits were reported for spatial working memory (67).

The Brain Body Life for Cognitive Decline (BBL-CD) trial evaluated the effects of an 8-week multidomain diet, exercise, and cognitive training program against an education only program in older adults with subjective cognitive decline or MCI. The intervention showed positive effects on overall cognition (68).

Finally, the FINGER trial, tested a 2-year multidomain lifestyle intervention in older adults at increased risk of dementia. The intervention showed beneficial effects on overall cognition (56), which was the primary outcomes, as well as pre-specified individual cognitive domains, and several other secondary outcomes such as risk of cognitive and functional decline and risk of multimorbidity.

While the primary results of these trials are inconsistent, several *post-hoc* analysis reported beneficial effects in specific subgroups, emphasizing the importance of targeting at-risk or selected populations and tailoring strategies to people with different risk profiles. Larger RCTs are, therefore, warranted to test such interventions (70). In this regard, the launch of initiatives such as the World-Wide FINGERS (WWFINGERS) (71), global network for dementia prevention trials, born from the experience of the FINGER trial, is a promising example. WWFINGERS currently includes about 40 countries and aims to test and implement FINGER-like interventions with specific cultural adaptations across diverse geographical and cultural settings and through a novel approach for resource sharing, data harmonization, and joint planning.

### The Need for Surrogate Outcomes in Multidomain Trials

Given the slow onset of neurodegenerative disorders, incident dementia, and cognitive impairment are unlikely to be suitable outcomes in the usually short timeframe (e.g., 2–3 years) of lifestyle-based intervention trials delivered to relatively fit, albeit at-risk, populations. Cognitive function measured e.g., through a neuropsychological battery covering various cognitive domains and sensitive to subtle age- and neuropathology-related changes is currently used as surrogate outcome in cognitively normal older adults (56) or MCI patients (68).

In the context of dementia risk reduction, tools specifically aimed to risk estimation, such as dementia risk scores, may be also considered as a plausible alternative for surrogate outcomes. Dementia risk scores can potentially be more sensitive to change than cognitive outcomes in middle-aged populations, long before the onset of the symptomatic phase of cognitive decline or dementia. Furthermore, their assessment is generally less demanding than that of neuropsychological tests measuring cognitive function, making their application in the context of large-scale intervention more feasible and a good complementary assessment.

The use of dementia risk scores as surrogate outcomes in dementia prevention trials is still very limited but nonetheless a subject of active investigation. The Body Brain Life trial was the first to use a dementia risk as primary outcome (19). Results showed that, in middle-aged adults with multiple risk factors, the multidomain intervention reduced the Australian National University-Alzheimer's Disease Risk Index (ANU-ADRI), a validated score for AD risk (19). A similar intervention, delivered in a primary care setting through eHealth, confirmed a significant reduction of the ANU-ADRI risk score in the 62 weeks of follow-up (72). The suitability of the CAIDE risk score as surrogate outcomes has been also investigated in *post-hoc* analyses of the first three, large dementia prevention multidomain intervention trials, FINGER, MAPT, and preDIVA, with promising results (23, 73).

### Implementation Policies and Link With Other NCDs

Implementing multidomain interventions may be the most effective way to increase the health of whole populations (70, 74). Many of the lifestyle risk/protective factors for dementia overlap with other non-communicable diseases (NCDs, e.g., *nutrition, tobacco consumption/cessation*, and *physical activity*), and several NCDs are also risk factors for dementia (e.g., CVD). Interventions targeting multiple factors across multiple NCDs are likely to lead to the most cost-effective and sustainable solutions. For example, primary prevention policies for dementia can be built onto existing programmes or campaigns related to other NCDs, including population health promotion in the areas of increased physical activity, healthy diet, tobacco cessation, management of weight, and alcohol use disorders, as well as specific strategies addressing cognitive stimulating activities, socially active lifestyles, and adequate childhood or formal education. The WHO Global Action Plan on the public health response to dementia 2017–2025 encourages this multi-sectorial approach in response to dementia, including alignment of dementia prevention activities to existing NCDs, mental health, and aging efforts world-wide. Researchers, policy makers and health professionals should be encouraged to develop, deliver, and promote evidence-based multidomain interventions at the population, community, and individual level that target both common and dementia specific risk factors when treating NCDs.

## Conclusions

The development of the first WHO guidelines for the risk reduction of cognitive decline and dementia through a rigorous review of the best quality evidence available (2) was a landmark in the field of dementia risk reduction. A number of recommendations for future research directions have emerged from it (Box 1), pointing in particular to the notion that, in this context, “one size does not fit all,” and therefore, multidomain approaches that can adapt to different populations and individuals, in terms of content, delivery, and timing, are more likely to be the most effective. Harmonization in trial design, with a specific focus on the choice of the most appropriate outcome measures and sustainability in large at-risk populations in the context of other chronic disorders have also emerged as key elements.

Box 1 **Recommendations for future research direction aimed a dementia risk reduction.**

Identify the ***population with the highest potential for improvement***, both in terms of individual benefit and number of people who can benefit

***Multidomain*** approach: targeting more risk/protective factors at once

***Tailored*** approach: flexible intervention characterized by a core of common principles which are adapted individually based on what is realistically achievable by each participant in terms of lifestyle changes

Explore the validity and potential use of ***surrogate outcomes*** (e.g., risk scores)

***Prospective harmonization*** of trial design, especially concerning the intervention design and outcome measures

Identify strategies to support post-trial ***sustainability***

Identify the most appropriate ***time to act*** within the life and disease course

Identify ***optimal intensity levels*** for each intervention

Investigate more closely the link between interventions and ***specific bran pathologies***

Investigate the effect of ***genetic risk, sex, and ethnic differences*** in modifying the response to preventive interventions

Optimize the implementation of interventions ***in the context of other NCDs***

The field of dementia prevention through lifestyle-based non-pharmacological interventions is still in its early phase and, compared to other age-related disorders (e.g., diabetes and falls), has been the subject of a relatively small number of large RCTs. Many opportunities and lines of investigation are open and, as highlighted by the recently published 2020 report of the Lancet commission for Dementia prevention, intervention and care (48), filling the current gaps in the knowledge related to dementia prevention will have a substantial impact in the healthcare of populations worldwide.

## Author Contributions

RS, MB, RP, AS, KA, and MK conceived and designed the study. RS, MB, and RP drafted the manuscript. NE, LZ, and JL wrote sections of the manuscript. All authors contributed to the revision of the manuscript, read, and approved the submitted version.

## Funding

This work was supported by the European Research Council (ERC) (Grant No. 804371), the Academy of Finland (Grant Nos. 287490, 319318, 317465, and 335524), the European Union Joint Programme—Neurodegenerative Disease (JPND) project EURO-FINGERS (Academy of Finland Grant No. 334804), Stiftelsen Stockholms Sjukhem, Center for Innovative Medicine (CIMED) at Karolinska Institutet, Knut and Alice Wallenberg Foundation, the Swedish Research Council for Health, Working Life and Welfare, Alzheimerfonden, Region Stockholm grants (ALF, NSV), Konung Gustaf V:s och Drottning Victorias Frimurarstiftelse, the Finnish Cultural Foundation, the National Health and Medical Research Council (NHMRC) (Grant Nos. 1102694 and 1100579), the NHMRC Dementia Centre for Research Collaboration, the Australian Research Council (ARC) (Grant No. FL19000011), Public Health England, and the Center for Disease Control and Prevention, United States of America.

## Author Disclaimer

The authors alone are responsible for the views expressed in this article and they do not necessarily represent the views, decisions or policies of the institutions with which they are affiliated.

## Conflict of Interest

KA is an advisor to Staying Sharp. The remaining authors declare that the research was conducted in the absence of any commercial or financial relationships that could be construed as a potential conflict of interest.

## Publisher's Note

All claims expressed in this article are solely those of the authors and do not necessarily represent those of their affiliated organizations, or those of the publisher, the editors and the reviewers. Any product that may be evaluated in this article, or claim that may be made by its manufacturer, is not guaranteed or endorsed by the publisher.
