# Supplementary material for: Development of the First WHO Guidelines for Risk Reduction of Cognitive Decline and Dementia: Lessons Learned and Future Directions
Source: Front Neurol. 2021 Oct 26;12:763573. doi: 10.3389/fneur.2021.763573 (PMC8577650; doi:10.3389/fneur.2021.763573)
Supplement: Supplementary file 1 [file Table_1.docx]

**Supplementary Table 1. Examples of studies included in the guidelines and more recent evidence published after the guidelines development process**

| **Risk factor** | **Evidence type** | | **Study** | | **Intervention/Exposure** | | **Outcome** | | **Effect parameter** | | **Effect size** | | **Confidence interval** | | **Heterogeneity assessment** | | **Reference** | |
| --- | --- | --- | --- | --- | --- | --- | --- | --- | --- | --- | --- | --- | --- | --- | --- | --- | --- | --- |
| **Physical Activity** | RCT | | SR/MA | | Aerobic exercise in normal cognition | | Global cognition (various tests) | | SMD | | 0.86 | | 0.24 to 1.47 | | N/A^a^ | | Barha et al., 2017 *Front. Neuroendocrinol.* | |
|  | RCT | | SR | | Aerobic exercise in MCI | | Global cognition (various tests) | | SMD | | 0.58 | | 0.18 to 0.98 | | - | | Song et al., 2018 *Int. J. Nurs. Stud.* | |
| **Tobacco consumption/cessation** | OBS | | SR | | Current smoking | | Incident Dementia | | RR | | 1.37 | | 1.23 to 1.52 | | - | | Beydoun et al., 2014 *BMC Public Health* | |
| **Nutrition** | RCT | | SR | | Medi Diet | | Global cognition | | SMD | | 0.24 | | 0.00 to 0.47 | | - | | Radd-Vagenas et al., 2018 *Am. J. Clin. Nutr.* | |
| **Alcohol use disorders** | OBS | | SR/MA | | High vs low beer consumption | | Incident dementia | | RR | | 1.84 | | 1.01 to 3.34 | | I^2^ 0.0% | | Xu et al., 2017 *Eur. J. Epidemiol.* | |
|  | OBS | | SS | | Frequent vs infrequent alcohol consumption at midlife | | Incident dementia | | OR | | 2.57 | | 1.19 to 5.52 | | - | | Anttila et al., 2004 *Br. Med. J*. | |
| **Cognitive activity** | RCT | | SR | | CognitiveTraining in healthy older adults | | Cognitive function (various tests) | | SMD | | 0.42 | | 0.21 to 0.63 | | - | | Chiu et al., 2017 *PLoS One* | |
|  | RCT | | SR/MA | | CognitiveTraining in MCI | | Cognitive function (various tests) | | SMD | | 0.22 | | 0.08 to 0.36 | | I^2^ 22.83% | | Sherman et al., 2017 *Neuropsychol. Rev.* | |
|  | OBS | | SR/MA | | Cognitive Engagement | | AD diagnosis | | RR | | 0.53 | | 0.42 to 0.63 | | I^2^ 90.5 % | | Anstey et al., 2019, *J. Alzheimer’s Dis.* Xu et al., 2015 *J. Neurol. Neurosurg. Psychiatry* | |
| **Overweight/Obesity** | RCT | | SR/MA | | Behavioural and lifstyle interventions for weight reduction | | Cognition Attention^b^ | | SMD | | 0.44 | | 0.26 to 0.62 | | I^2^ 60 % | | Veronese et al., 2017 *Neurosci. Biobehav. Rev.* | |
|  | OBS | | SR/MA | | Midlife overweight/obesity (age < 65) | | Incident dementia | | RR | | 1.41 | | 1.20 to 1.66 | | N/A^c^ | | Pedditizi et al., 2016 *Age Ageing* | |
|  |  |  |  |  | Late life overweight/obesity (age ≥ 65) | | Incident dementia | | RR | | 0.83 | | 0.74 to 0.94 | | I^2^ 53 %^d^ | |  |  |
| **Hypertension** | RCT | | SR/MA | | Pharmacological treatment | | Cognitive function (various texts) | | RR | | 0.96 | | 0.87 to 1.06 | | N/A | | Parsons et al., 2016 *Future Cardiol.* | |
|  | RCT | | SR/MA | | Pharmacological treatment in SPRINT-MIND | | Dementia/Cognitive Impairment | | OR | | 0.93 | | 0.88 to 0.98 | | I^2^ 0.0 % | | Hughes et al., 2020^e^  *JAMA - J. Am. Med. Assoc.* | |
|  | RCT | | SR/MA | | Lifestyle and/or pharmacological interventions | | Incident Dementia | | RR | | 0.94 | | 0.76 to 1.18 | | I^2^ 0.0 %^f^ | | van Middelaar et al., 2018^e^ *Stroke* | |
|  | OBS | | SR | | Hypertension, high DBP/SBP | | AD diagnosis | | RR | | 0.98 to 2.38 | | 0.8 to 1.19; 1.34 to 4.23 | | - | | Anstey et al., 2019^e^  *J. Alzheimer’s Dis.* | |
|  | OBS | | SR | | Antihypertensives | | AD diagnosis | | RR | | 0.71 to 0.92 | | 0.59 to 0.83; 0.79 to 1.08 | | - | | Anstey et al., 2019^e^  *J. Alzheimer’s Dis.* | |
| **Diabetes mellitus** | RCT | | SR/MA | | Pharmacological treatment | | Global cognition (MMSE)^g^ | | MD | | -0.00 | | -0.08 to 0.07 | | N/A | | Areosa et al., 2017 *Cochrane Database Syst. Rev.* | |
|  |  |  |  |  |  |  | MMSE score reduction ≥ 3 | | OR | | 0.98 | | 0.88 to 1.08 | | N/A | |  |  |
|  | RCT | | SS | | Lifestyle interventions | | Global cognition | | SMD | | -0.014 | | -0.04 to 0.01 | | - | | Rapp et al., 2017 *J. Am. Geriatr. Soc.* | |
|  | RCT | | SS | | Lifestyle interventions | | Global cognition (3MS)**^h^** | | SMD | | 0.114 | | 0.007 to 0.222 | | - | | Espeland et al., 2017 *Neurology* | |
|  | OBS | | SR | | Diabetes | | AD diagnosis | | RR | | 1.33 to 1.57 | | 1.14 to 1.52; 1.41 to 1.75 | | - | | Anstey et al., 2019^e^  *J. Alzheimer’s Dis.* | |
| **Dyslipidaemia** | RCT | SR/MA | | Statin treatment | | Incident Dementia | | OR | | 1 | | 0.61 to 1.65 | | N/A^i^ | | McGuinness et al., 2015 *Cochrane Database Syst. Rev.* | |  |
|  | OBS | SR/MA | | Statins use | | Incident dementia | | RR | | 0.62 | | 0.43 to 0.81 | | I^2^ 70.8 % | | Song et al., 2013 *Geriatr. Gerontol. Int.* | |  |
| **Depression** | RCT | SR/MA | | Vortiotexine | | Cognitive function (DSST)**^j^** | | SMD | | 0.34 | | 0.18 to 0.49 | | N/A | | Baune et al., 2018 *Int.J. Neuropsychopharmacol.* | |  |
|  | OBS | SR | | Depression | | AD diagnosis | | RR | | 1.06 to 2.04 | | 1.02 to 1.10; 1.40 to 2.98 | | - | | Anstey et al., 2019^e^  *J. Alzheimer’s Dis.* | |  |

3ME: Modified Mini Mental State Exam; AD: Alzheimer's disease; DSST: digit symbol substitution test; MA: meta-analysis; MMSE: mini mental state examination; MD: mean difference; N/A: not available or not reported; OBS: observational study; OR: odds ratio; RCT: randomised controlled trial; RR: relative risk; SMD: standard mean difference; SR: systematic review; SS: single study.

a: Assessed but not reported

b: The effect on global cognition was not reported; attention was the cognitive domain for which the largest significant effect was reported.

c: Formal assessment of heterogeneity was not reported, but the author stated that no evidence of heterogeneity was found

d: Heterogeneity reported for obesity; for overweight the author stated that no evidence of heterogeneity was found

e: New evidence published after the conclusion of the guidelines’ development process

f: It includes two studies, one combining pharmacological and lifestyle interventions.

g: Folstein, M.F., Folstein, S.E., McHugh, P.R., 1975. “Mini-mental state”. A practical method for grading the cognitive state of patients for the clinician. J. Psychiatr. Res. 12, 189–198.

h: Teng, E.L., Chui, H.C., 1987. The Modified Mini-Mental State (3MS) examination. J. Clin. Psychiatry 48, 314–318.

i: Only one study included

j: Wechsler D., 1939. The Measurement of Adult Intelligence. The Williams & Wilkins Company, Baltimore, MD.
